# A PAX3/BRN2 rheostat controls the dynamics of BRAF mediated MITF regulation in MITF^high^/AXL^low^ melanoma

**DOI:** 10.1111/pcmr.12741

**Published:** 2018-10-19

**Authors:** Michael P. Smith, Sareena Rana, Jennifer Ferguson, Emily J. Rowling, Keith T. Flaherty, Jennifer A. Wargo, Richard Marais, Claudia Wellbrock

**Affiliations:** ^1^ Manchester Cancer Research Centre, Faculty of Biology, Medicine & Health, Division of Cancer Sciences The University of Manchester Manchester UK; ^2^ Division of Molecular Pathology The Institute of Cancer Research London UK; ^3^ Massachusetts General Hospital Cancer Center Boston Massachusetts; ^4^ Divison of Surgical Oncology University of Texas MD Anderson Cancer Center Houston Texas; ^5^ Molecular Oncology Group Cancer Research UK Manchester Institute, The University of Manchester, Astra Zeneca Logistics Centre Macclesfield UK

**Keywords:** AXL, BRAF, BRN2, MEK, MITF, PAX3

## Abstract

The BRAF kinase and the MAPK pathway are targets of current melanoma therapies. However, MAPK pathway inhibition results in dynamic changes of downstream targets that can counteract inhibitor‐action not only in during treatment, but also in acquired resistant tumours. One such dynamic change involves the expression of the transcription factor MITF, a crucial regulator of cell survival and proliferation in untreated as well as drug‐addicted acquired resistant melanoma. Tight control over MITF expression levels is required for optimal melanoma growth, and while it is well established that the MAPK pathway regulates MITF expression, the actual mechanism is insufficiently understood. We reveal here, how BRAF through action on the transcription factors BRN2 and PAX3 executes control over the regulation of MITF expression in a manner that allows for considerable plasticity. This plasticity provides robustness to the BRAF mediated MITF regulation and explains the dynamics in MITF expression that are observed in patients in response to MAPK inhibitor therapy.

**SIGNIFICANCE**

The BRAF and the MAPK pathway are targets of current melanoma therapies, but drug tolerance develops through adaptive mechanisms such as MITF upregulation. During melanoma development, BRAF induces expression of MITF in order to control tumour growth, but this regulation is inverted when BRAF is inhibited. We identify a mechanism involving BRN2 and PAX3 that provides a biologically required robustness to the BRAF induced MITF expression, and that explains the MITF dynamics that are observed after MAPK inhibition in patients. Our data suggest that monitoring the dynamics of the identified molecular players could contribute to improved control over therapy response.

## 1 INTRODUCTION

The serine threonine kinases BRAF and MEK are major regulators of the ERK/MAP kinase pathway, which is deregulated in the majority of melanomas. MEK is hyper‐activated in over 90% of melanomas, and BRAF harbours activating mutations in approximately 50% of melanomas (Wellbrock & Arozarena, 2016). These deregulations reflect the crucial role of BRAF and the MAPK pathway in governing melanoma cell survival and proliferation. In fact, in melanoma cells the MAPK pathway has assumed control over the function and expression of MITF, the master transcriptional regulator of genes that not only define the melanocytic lineage, but also drive cell cycle progression and survival (Kundu, Quirit, Khouri, & Firestone, 2017; Wellbrock & Arozarena, 2015; Wellbrock et al., 2008). In line with MITF being a survival regulator, we and others have shown that overexpression of MITF limits the efficacy of BRAF and MEK inhibitors (Haq et al., 2013; Muller et al., 2014; Smith et al., 2013, 2017 ). Furthermore, MITF is required for the survival of drug‐addicted resistant melanomas (Kong et al., 2017) and MITF upregulation is found in up to 23% of melanomas progressed on treatment (Smith et al., 2016; Van Allen et al., 2014). Thus, the fact that the MAPK pathway regulates MITF expression and function is critical, considering that melanoma patients are treated with BRAF and MEK inhibitors (MAPKi).

However, findings regarding the mechanisms underlying MITF regulation by the MAPK pathway suggest a significant degree of complexity. Phosphorylation by ERK regulates MITF function and degradation (Wu et al., 2000), but the relevance of the so far identified ERK‐phosphorylation sites is still a matter of debate (Bauer et al., 2009; Wellbrock & Marais, 2005). At transcriptional level BRAF induces expression from a proximal region within the *MITF* promoter through BRN2, a POU domain transcription factor which positively regulates MITF expression in many melanoma cell lines (Cook, Smith, Smit, Leonard, & Sturm, 2005; Kundu et al., 2017; Simmons, Pierce, Al‐Ejeh, & Boyle, 2017; Thurber et al., 2011; Wellbrock et al., 2008). Intriguingly, however, independently of BRAF, BRN2 has also been shown to act as suppressor of MITF (Goodall et al., 2008; Kobi et al., 2010), and as such BRN2 expression has been predicted to correlate with a dedifferentiated phenotype with low MITF expression, which is also called the “AXL^high^” phenotype (Tirosh et al., 2016).

Another transcriptional regulator of MITF, PAX3 is also regulated by the MAPK pathway (Smith et al., 2016). However, in contrast to BRN2, PAX3 expression is suppressed by BRAF, and MAPKi induce an upregulation of PAX3 and subsequently MITF expression (Smith et al., 2016). Intriguingly, while this upregulation is found in tumours from patients on treatment with MAPKi (Rambow et al., 2018; Smith et al., 2016), other studies report reduction of MITF RNA levels in response to MAPKi (Johannessen et al., 2013; Kono et al., 2006). This suggests that the regulation of MITF expression by the MAPK pathway is highly complex, and multiple factors contribute to the dynamics of MITF levels downstream of ERK in melanoma cells.

With MITF's central role in melanoma and its link to the MAPK pathway, it is crucial to fully understand the impact that MAPKi have on MITF during treatment. Here, we identify a mechanism that leads to crucial plasticity in the regulation of MITF, and that explains controversial observations made with regard to MAPK inhibition.

## 2 MATERIALS & METHODS

### 2.1 Cell culture

Melanoma cell lines (details in Supporting Information) were grown in DMEM/10% FCS (PAA, Yeovil, UK). PD184352, AZD6244, U0126 and vemurafenib were from Selleck Chemicals (Newmarket, UK).

### 2.2 RNA isolation and qPCR and expression data analysis

RNA from cell lines and tumours (Smith et al., 2016) was isolated with TRIZOL^®^ and selected genes were amplified by quantitative real‐time PCR. Data sets were analysed in and exported from Oncomine (Compedia Bioscience, Ann Arbor, MI). qPCR primers and siRNA sequences are described in Supporting Information.

### 2.3 Cell lysis and immunoprecipitation

Cells were lysed in SDS sample buffer or 1% Triton‐X100 buffer and analysed by Western blotting as described (Wellbrock & Schartl, 2000). BRN2 was precipitated from 1 mg of total protein from WM266‐4 cells 48 hr after transfection with pEF‐PAX3, using 2 µg of BRN2‐antibody (B‐2). PAX3 was precipitated from 4 mg of total protein from WM164 cells using 4 µg of PAX3‐antibody (C‐20). Details of antibodies are listed in Supporting Information.

### 2.4 Chromatin immunoprecipitation assay

Chromatin immunoprecipitation (ChIP) assays were performed using Quick ChIP (Novus Biologicals, Abingdon, UK) and anti‐PAX3 antibodies (details listed in Supporting Information). The primers used for PCR were 5′‐CGTCACTTAAAAAGGTACCTTTATATTTATG‐3′ and 5′‐TGTTTTAGCTAGCACCAATCCAGTGAGAGACGG‐3′ for *MITF* and 5′‐AACAAAACCAAT‐TAGGAACCTT‐3′ and 5′‐ATTTCCTTCATCTTGTC‐CTTCT‐3′ for *CYCLIND1*.

### 2.5 Luciferase reporter assays

The pGL2‐*M‐MITF*‐333 promoter construct and the pEFmBRAF^V600E^ expression construct have been described (Wellbrock et al., 2008). A375 cells were transfected with 0.6 µg of reporter plasmid, 0.3 µg of expression plasmid and 0.3 µg of pSV‐β‐Galactosidase (Promega, Madison, WI, USA); cells were analysed as described (Wellbrock et al., 2008).

### 2.6 Statistical analysis

If not indicated otherwise, data represent the results for assays performed in triplicate, with error bars to represent SEM. Statistics used were: Student's *t* test and One‐way ANOVA with Tukey's post hoc test performed using GraphPad Prism version 7.00 for Mac OS (GraphPad Software, San Diego CA, USA). Pearson correlation was used to analyse associated gene expression.

## 3 RESULTS

### 3.1 ***PAX3, but not BRN2 correlates with MITF expression also in AXL^high^/MITF^low^*** ***cells***

**Figure 1:**
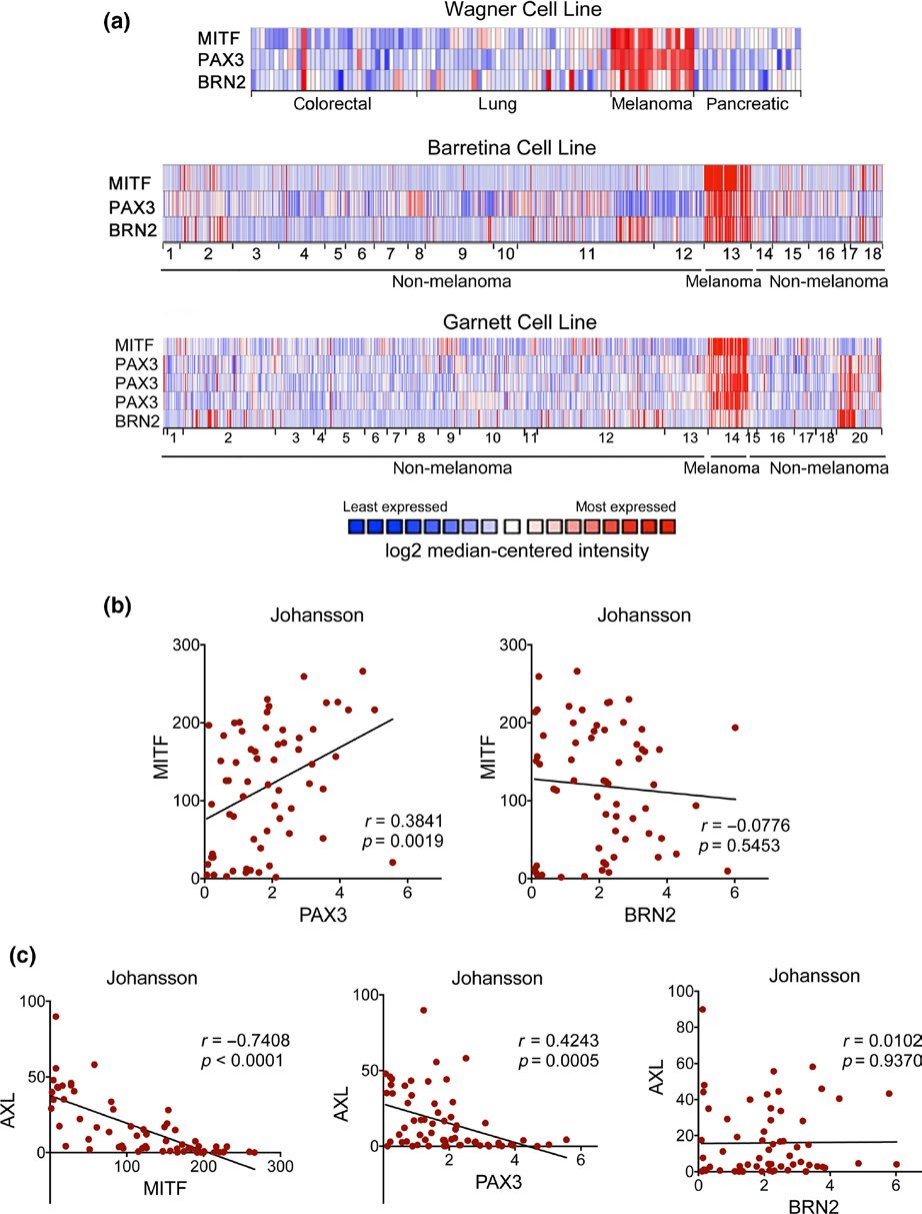
BRN2 expression does not correlate with the AXL high/MITF‐low phenotype. (a) Expression of MITF, PAX3, and BRN2 in Oncomine Cell line datasets Wagner (Wagner et al., 2007), Barretina (Barretina et al., 2012) and Garnett (Garnett et al., 2012). For Barretina: 1. Bladder, 2. Brain/CNS, 3. Breast, 4. Colon, 5. Oesophagus, 6. Gastric, 7. Head and Neck, 8. Kidney, 9. Leukaemia, 10. Liver, 11. Lung, 12. Lymphoma, 13. Melanoma, 14. Myeloma, 15. Ovary, 16. Pancreas, 17. Prostate, 18. Bone and for Garnett: 1. Bladder, 2. Brain/CNS, 3. Breast, 4. Cervix, 5. Colon, 6. Oesophagus, 7. Gastric, 8. Head and Neck, 9. Kidney, 10. Leukaemia, 11. Liver, 12. Lung, 13. Lymphoma, 14. Melanoma, 15. Myeloma, 16. Other, 17. Ovary, 18. Pancreas, 19. Prostate, 20. Bone. (b) Pearson correlation analysis of MITF expression with PAX3 and BRN2 in the Johansson dataset. (c) Pearson correlation analysis of AXL expression with MITF, PAX3, and BRN2 in the Johansson dataset

Reflecting their cancer specific relevance, expression of MITF, PAX3, and BRN2 is significantly enriched in melanoma cell lines as seen in a panel covering >20 general cancer types (Barretina et al., 2012; Garnett et al., 2012; Wagner et al., 2007) (Figure 1a). PAX3 is a crucial regulator of MITF in the melanocyte lineage, and its expression displayed significant positive correlation with MITF levels (Supporting Information Figure S1A). This was independent of the *BRAF*/*NRAS* mutation status (Supporting Information Figure S1A and B), and suggests that PAX3 induced MITF expression is an inherent melanocyte lineage trait that is conserved in melanoma independently of the genetic background.

Despite the enrichment of BRN2 expression in melanoma cells, and the established link of BRN2 to the regulation of MITF expression (Goodall et al., 2008; Wellbrock et al., 2008), the correlation of BRN2 and MITF mRNA levels was not significant in all data sets (Figure 1b and Supporting Information Figure S1B). This was independent of *BRAF*/*NRAS* mutations (not shown), and suggested that the regulation of MITF by BRN2 is more complex and dependent on additional factors.

Thus, we considered individual melanoma phenotypes linked to distinct MITF expression levels (Hoek et al., 2006; Tirosh et al., 2016). Thereby, MITF expressing cells define the “MITF^high^” phenotype, in which MITF governs a “proliferation and differentiation” gene‐expression signature. On the other hand, when MITF expression is minimal, this marks a population of MITF^low^ cells, which are dedifferentiated with regard to the melanocyte lineage. If this coincides with the expression of AXL, it defines the “AXL^high^/MITF^low^ invasive” phenotype (Konieczkowski et al., 2014). Considering the MITF^high^ and AXL^high^ phenotypes in our analysis, we confirmed inverse correlation of MITF with AXL expression, and importantly PAX3 followed the same trend (Figure 1c and Supporting Information Figure S1C). The situation was, however, different with BRN2, whose expression did not significantly correlate with AXL (Figure 1c and Supporting Information Figure S1D). Thus, although BRN2 has been established as driver of an invasive phenotype classified by low MITF expression (Arozarena et al., 2011; Fane, Chhabra, Smith, & Sturm, 2018; Goodall et al., 2008), its expression pattern does not align with the marker for the well‐characterized AXL^high^/MITF^low^ phenotype.

### 3.2 BRN2 and PAX3 exhibit inverse expression patterns in MITF^high^ melanoma cells

**Figure 2:**
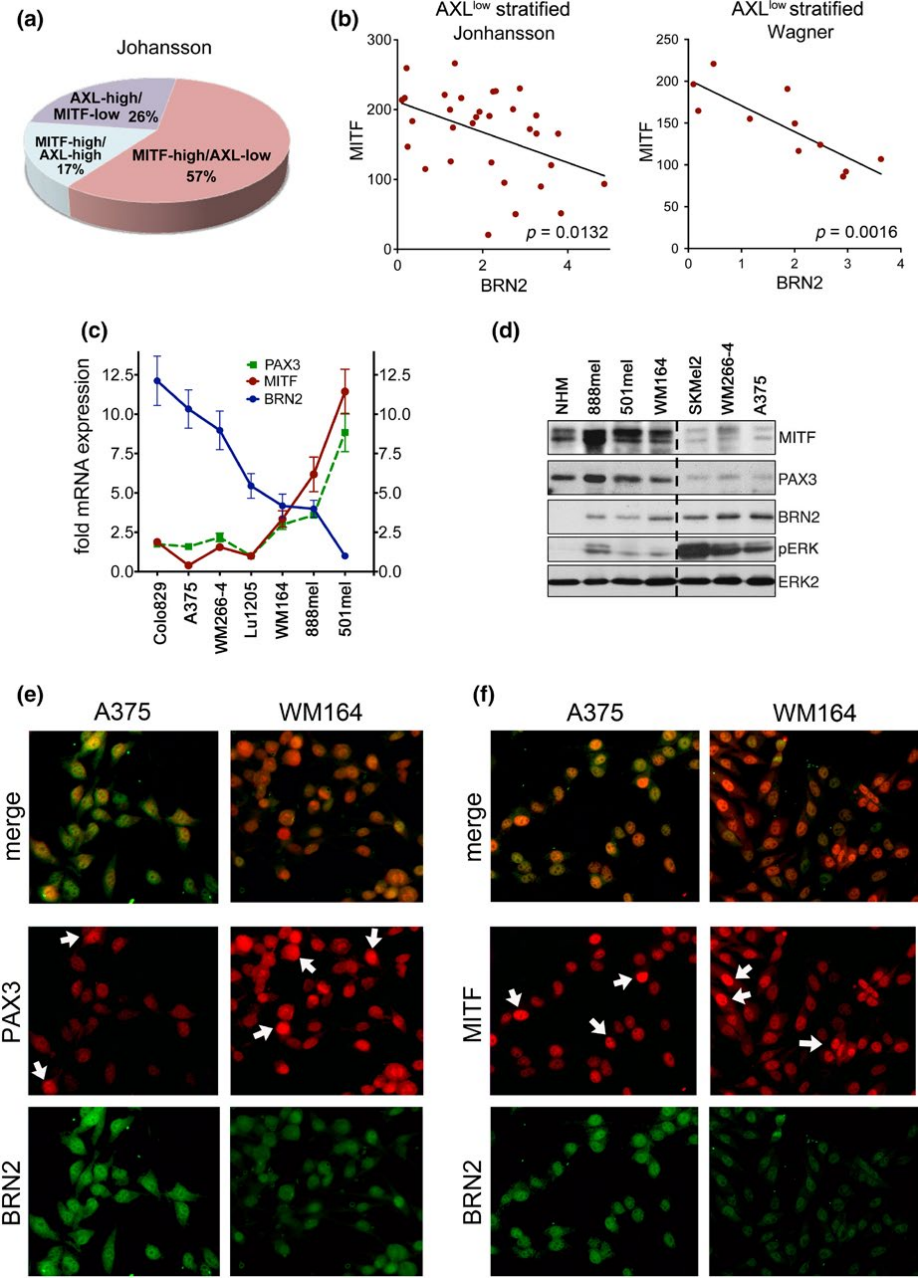
BRN2 and PAX3 expression patterns in MITF‐high melanoma cells. (a) Analysis for AXL and MITF expression in the Johansson dataset. (b) Pearson correlation analysis of MITF expression with BRN2 in MITF‐high cells. (c) Relative mRNA expression of MITF, PAX3, BRN2. PAX3 expression of Lu1205 cells was set 1; BRN2 expression in 501mel cells was set 1. (d) Western blot analysis of the indicated melanoma cell lines for MITF, PAX3, BRN2, pERK, and ERK2. (e) Immunofluorescence analysis for BRN2 and PAX3 in A375 and WM164 cells. (f) Immunofluorescence analysis for BRN2 and MITF in A375 and WM164 cells

Because BRN2 was not linked to the AXL^high^ population, we stratified for the AXL^low^ population, because its expression programme is governed by MITF, the actual target of BRN2. Mirroring what is observed in melanoma biopsies (Sensi et al., 2011; Tirosh et al., 2016), ~57% of melanoma lines in the Johansson dataset (Johansson, Pavey, & Hayward, 2007) fall into the MITF^high^/AXL^low^ group (Figure 2a). Strikingly, in this MITF^high^/AXL^low^ population the correlation of BRN2 with MITF was now significant, but inverse, and the same was seen in the other data sets (Figure 2b and Supporting Information Figure S2A).

We could confirm the correlation of MITF with BRN2 and PAX3 expression at RNA level (Figure 2c) and at protein level (Figure 2d) in a panel of melanoma cell lines. As previously seen (Cook et al., 2003), melanocytes (NHM) did not express BRN2 when ERK was not activated. However, in melanoma cells we saw an increase in BRN2 protein at higher levels of ERK phosphorylation (Figure 2d). Likewise, the ERK activity surrogate marker *DUSP6* displayed a positive correlation with BRN2 in all data sets (Supporting Information Figure S2B). In contrast, PAX3 expression displayed a negative correlation with ERK phosphorylation (Figure 2d). This is entirely in agreement with our finding that active ERK triggers the suppression of PAX3 through upregulation of SKI (Smith et al., 2016).

At single‐cell level, BRN2 was more evenly expressed throughout individual cell populations, but PAX3 expression appeared more heterogeneous (Figure 2e). Intriguingly, the analysis for MITF expression revealed a similar heterogeneity, and while BRN2 was co‐expressed with MITF it did not display the same heterogeneity (Figure 2f).

### 3.3 PAX3 is required for BRAF^V600E^ mediated MITF expression

**Figure 3:**
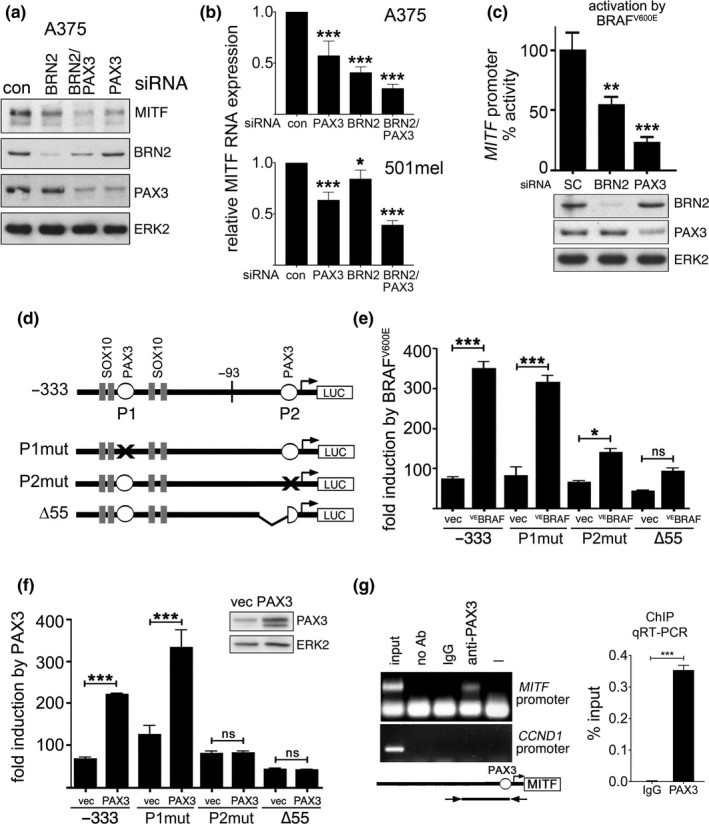
BRAF regulates MITF expression through PAX3. (a) Western blot for MITF, BRN2, PAX3, and ERK2 of A375 cells treated with the indicated siRNAs. (b) qRT‐PCR analysis of MITF expression in A375 and 501mel cells treated with the indicated siRNAs. (c) Luciferase assay for *M‐MITF* promoter activity in A375 cells co‐transfected with control, PAX3 or BRN2 siRNAs and a ^V600E^BRAF expression plasmid. Western blotting confirmed the knock down for PAX3 and BRN2. (d) Schematic of the *M‐MITF* promoter (−333/+120) with potential PAX3 binding sites (P1, P2) and respective mutations. (e) Luciferase assay for the WT or mutated *M‐MITF* promoter (−333/+120) in A375 cells transfected with a ^V600E^BRAF and (f) with a PAX3 expression plasmid. (g) Chromatin immunoprecipitation (ChIP) from A375 (left) and WM164 (RT‐qPCR, right) cells using PAX3 specific antibodies or IgG. Primers amplify a region from −170 to +120 of the *M‐MITF* promoter. In A375 cells, the *cyclin D1* promoter was analysed as control. Data presented as the mean ± SEM are from at least three biological repeats

Using RNAi we confirmed previous findings (Cook et al., 2005; Simmons et al., 2017; Smith et al., 2016; Thurber et al., 2011; Wellbrock et al., 2008) that both, BRN2 and PAX3 contribute to MITF expression in melanoma cells (Figure 3a and b and Supporting Information Figure S3). BRN2 expression is induced by MAPK signalling and is used by BRAF^V600E^ to stimulate MITF expression (Kundu et al., 2017; Wellbrock et al., 2008). Because PAX3 regulates MITF expression, but its own expression is reduced through MAPK signalling, we wanted to assess its function downstream of BRAF. Intriguingly, we found that similar to BRN2, PAX3 strongly contributes to the BRAF^V600E^ induced transcription from the *MITF* promoter (Figure 3c).

The *MITF* promoter contains two putative PAX3 binding sites, P1 at −260/−244 and P2 at −40/−25 from the transcription start site (Bondurand et al., 2000). Only mutation (P2mut) or deletion (∆55) affecting the P2 site reduced the BRAF^V600E^ induced promoter activation (Figure 3d and e), confirming previous results that BRAF^V600E^ stimulates transcription within 93 bp upstream of the transcription initiation site (Wellbrock et al., 2008). Mutating (P2mut) or deleting (∆55) P2 also completely abolished the ability of PAX3 to stimulate transcription from the *MITF* promoter in melanoma cells (Figure 3f). By performing ChIP we could show that PAX3 bound the region covering the P2 site (Figure 3g), further supporting that this site, which is also important for the regulation by BRAF (Figure 3e) is used by PAX3 in melanoma cells.

### 3.4 PAX3 is essential for the BRAF/BRN2 mediated induction of MITF expression

**Figure 4:**
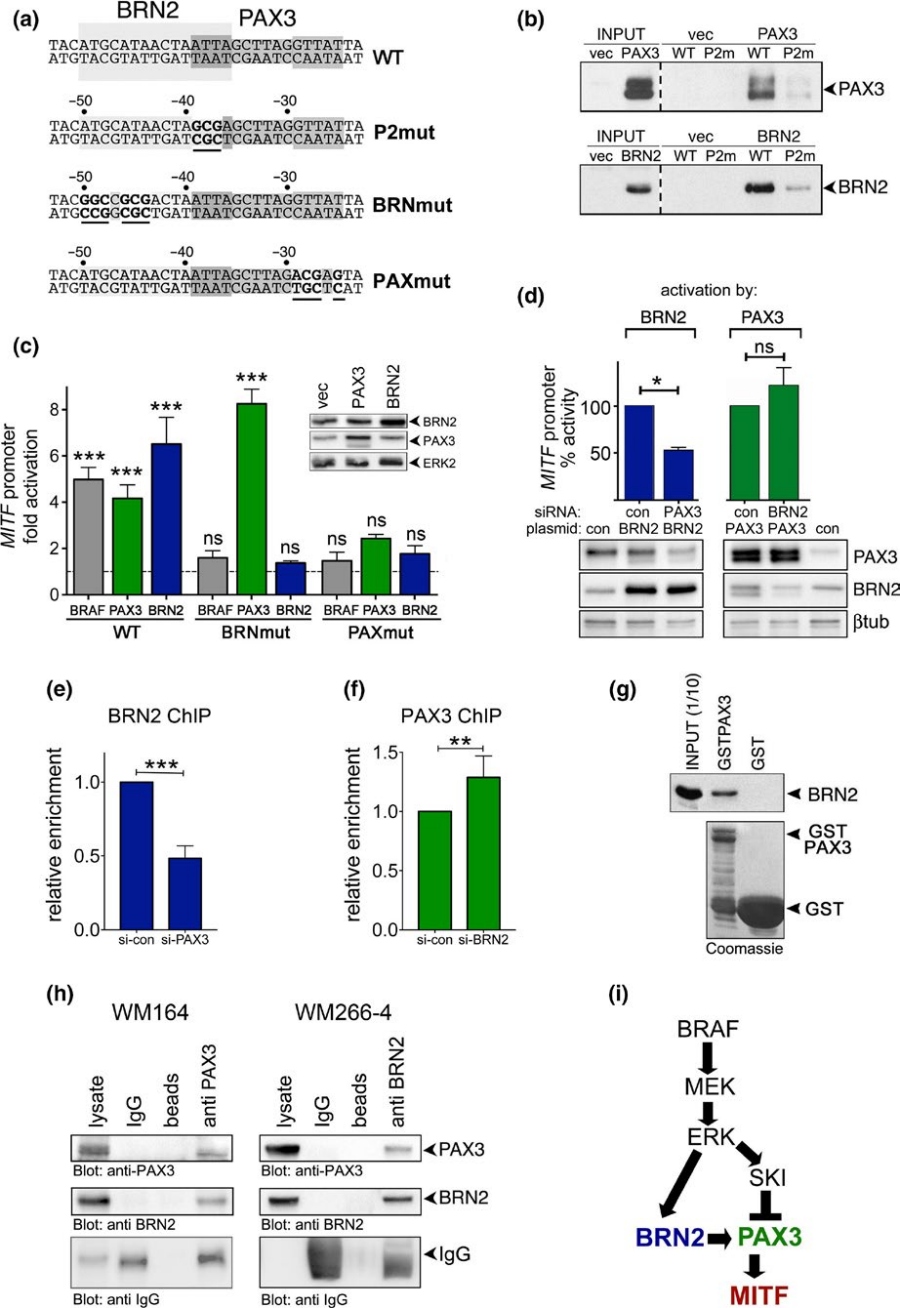
The BRAF/BRN2 mediated induction of MITF expression requires PAX3. (a) Sequence of the *M‐MITF* promoter (−53/−23) with BRN2 (−36/−51) and PAX3 (−25/−39) binding sites with respective mutations. (b) In vitro DNA binding. Cell extracts from Hela cells overexpressing PAX3 or BRN2 were incubated with the −77 to −20 region of the *MITF* promoter and bound proteins were analysed by Western blotting. (c) Luciferase assay for the WT or mutated *M‐MITF* promoter (−333/+120) in A375 cells transfected with ^V600E^BRAF, PAX3, or BRN2 expression plasmids. (d) Luciferase assay for the *M‐MITF* promoter (−333/+120) in A375 cells transfected with control, PAX3 or BRN2 siRNA and plasmids expressing either BRN2 or PAX3. (e) ChIP qRT‐PCR for BRN2 in WM164 cells transfected with control or PAX3 siRNA. (f) ChIP qRT‐PCR for PAX3 in WM164 cells transfected with control or BRN2 siRNA. (g) Western blot of GST pull down of endogenous BRN2. Extracts from A375 cells were incubated with immobilized GST or GSTPAX3, expression of which was analysed by Coomassie staining. (h) Co‐immunoprecipitation of PAX3 with BRN2 from WM164 cells or from ectopically PAX3 overexpressing WM266‐4 cells. (i) Proposed mechanism of regulation of MITF by BRAF. Data presented as the mean± SEM are from at least three biological repeats

The P2mut mutation clearly abrogated the ability of PAX3 to activate the *MITF* promoter, but this mutation also affects the BRN2 binding site (Figure 4a and b). We therefore analysed promoter mutants that allowed dissecting the individual contributions of both transcription factors more specifically. In line with our findings that BRAF uses BRN2 to drive MITF expression (Wellbrock et al., 2008), mutations specifically affecting the BRN2 binding site (BRNmut) decreased the BRN2 and the BRAF^V600E^ mediated promoter activation (Figure 4a and c). This mutation did not affect PAX3 activity (Figure 4a and c). Intriguingly, however, specifically mutating the PAX3 binding site (PAXmut) not only reduced PAX3 and BRAF^V600E^, but also BRN2 induced promoter activation (Figure 4a and c). Thus, PAX3 can act independently of BRN2, but BRN2 requires PAX3 for full activation. Indeed, while PAX3 can still activate the promoter when BRN2 is depleted, the ability of BRN2 to stimulate the *MITF* promoter is reduced after PAX3 knock down (Figure 4d). In line with this, depleting PAX3 reduced the binding of BRN2 to the *MITF* promoter (Figure 4e). Depleting BRN2 induced a slight increase in PAX3 binding (Figure 4f). These effects further support the idea that there is an interaction of the two transcription factors at the *MITF* promoter. In addition, PAX3 and BRN2 interact in vitro (Figure 4g) and importantly, this is also seen in cells (Figure 4h). Together our data suggest a mechanism in which both PAX3 and BRN2 contribute to *MITF* promoter activation downstream of BRAF^V600E^. Thereby BRN2 is the crucial link between BRAF and MITF, and uses PAX3 for full activation (Figure 4i). Importantly, in the context of oncogenic BRAF, MAPK signalling stimulates the expression of BRN2 (Wellbrock et al., 2008), but it suppresses PAX3 expression (Smith et al., 2016), and hence basal MITF expression levels are directly linked to active ERK downstream of BRAF.

### 3.5 A PAX3/BRN2 rheostat controls MITF expression downstream of BRAF

**Figure 5:**
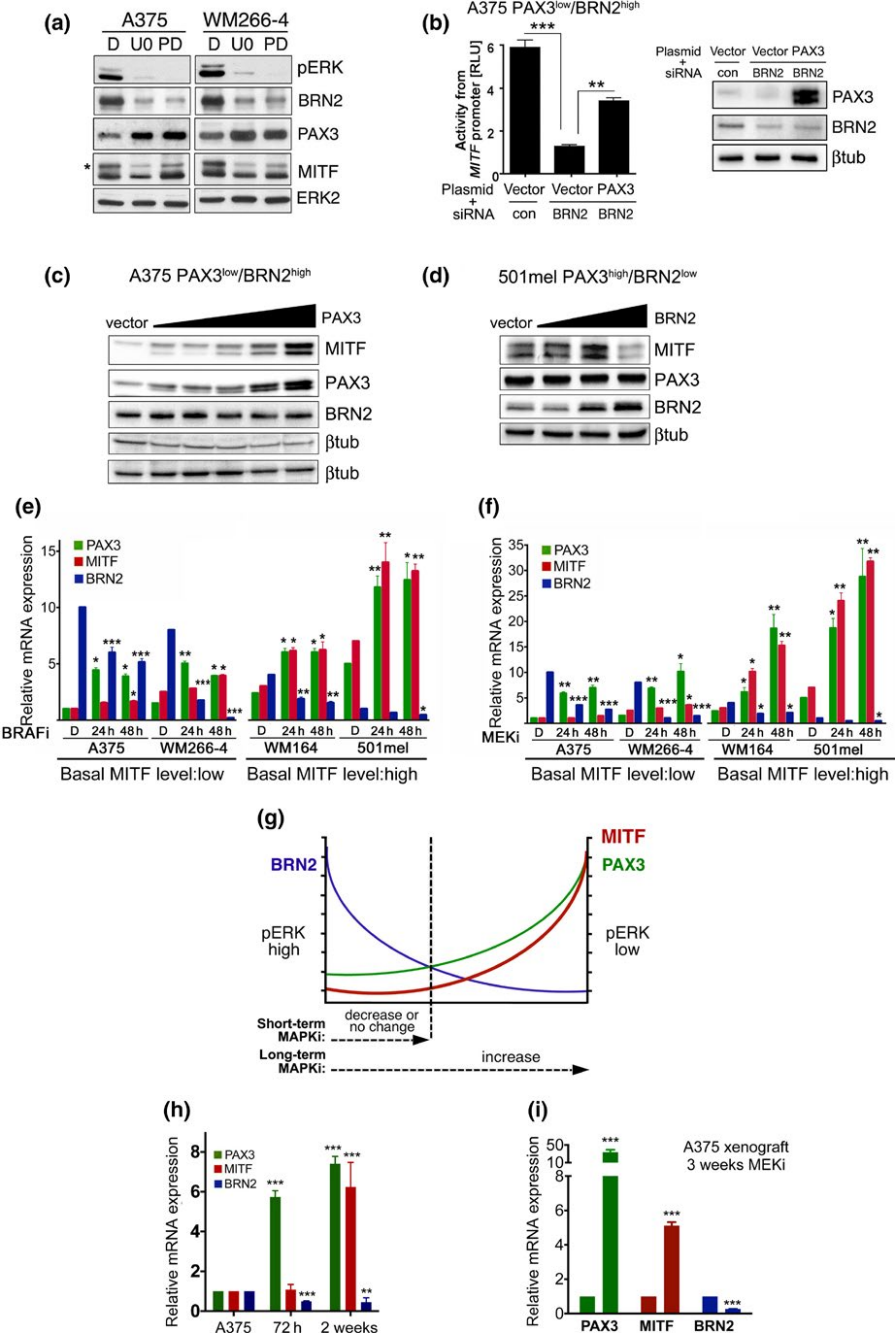
Long‐term BRAF and MEK inhibition increases PAX3 and MITF expression. (a) Western blot analysis for MITF, PAX3, BRN2, pERK and ERK2 in A375 and WM266‐4 cells treated with 10 µM U0126 (U0), 1 µM PD184352 (PD) or DMSO (D) for 24 hr. (b) Luciferase assay for the *M‐MITF* promoter activity in A375 cells co‐transfected with either control or BRN2 siRNAs and a PAX3 expression or control plasmid. (c) Western blot for the indicated proteins from A375 cells transfected with increasing amounts (200–600 ng) of a PAX3 expression plasmid. (d) Western blot for the indicated proteins from 501mel cells transfected with increasing amounts (300–500 ng) of a BRN2 expression plasmid. (e) RT‐qPCR analysis of *PAX3*, *MITF*, *BRN2 *expression in the indicated cell lines untreated or treated with 1 µM vemurafenib (BRAFi) or (f) with 1 µM AZZD6244 (MEKi). (g) Model for the regulation of MITF by PAX3 and BRN2. Short‐term MAP kinase pathway inhibition results in reduced BRN2 and increased PAX3 expression, but long‐term inhibition will lead to constant PAX3 upregulation and consequently increased MITF expression. (h) RT‐qPCR analysis of *PAX3*, *MITF*, *BRN2 *expression in A375 cells were treated with MEKi for the indicated times. (i) RT‐qPCR analysis for *PAX3*, *MITF*, *BRN2 *expression in A375 melanoma xenografts from mice treated with DMSO or with MEKi for 3 weeks. Data presented as the mean ± SEM are from at least three biological repeats

To test the role of active ERK in our model, we analysed the consequences of MAPK pathway inhibition for the PAX3/BRN2 rheostat. As expected, treating A375 and WM266‐4 cells with MEKi for 24 hr reduced BRN2 expression and increased PAX3 levels (Figure 5a). Nevertheless, MITF protein expression was not majorly altered apart from a reduction in the ERK‐phosphorylated form (*) (Figure 5a). It appears therefore that in these PAX3^low^/BRN2^high^ cell lines, where BRN2 is the main driver of MITF expression, upregulation of PAX3 can compensate for the reduction in BRN2. Indeed, overexpression of PAX3 can partially rescue the reduced *MITF* promoter activity seen after BRN2 depletion (Figure 5b). Thus, the relative amounts of PAX3 and BRN2 appear to control basal MITF expression levels downstream of oncogenic BRAF. Such a situation was observed when relative expression levels were changed by either overexpressing PAX3 in BRN2^high^ cells or BRN2 in PAX3^high^ cells (Figure 5c and d).

When BRAF or MEK were inhibited in PAX3^low^/BRN2^high^ cells (A375, WM266‐4), the overall changes to MITF transcripts over 48 hr were not major (Figure 5e and f), suggesting that the induced PAX3 upregulation simply compensated for the loss of BRN2. However, in PAX3^high^/BRN2^low^ cells (501mel, WM164), where MITF expression is predominantly driven by PAX3, BRAF/MEK inhibition led to an increase in MITF expression within 48 hr (Figure 5e and f). As such, we propose a model in which MEK/ERK activation downstream of BRAF is closely linked to BRN2/PAX3 driven MITF expression (Figure 5g). In this model, the level of ERK activation controls basal expression of PAX3 and BRN2, and the impact of short‐term MAPK pathway inhibition on MITF expression will depend on the PAX3/BRN2 ratio. This model can explain why in some cell lines MITF expression drops in the presence of MAPKi, whereas in others an increase is observed. Our model also suggests that prolonged upregulation of PAX3 expression, as response to long‐term MAPK pathway inhibition should increase MITF expression even in PAX3^low^ cells. Indeed, this was seen in PAX3^low^/BRN2^high^ A375 cells after 2 weeks of MEKi exposure (Figure 5h). A similar effect was observed in xenografts after long‐term exposure to MEK inhibitor (Figure 5i).

### 3.6 The PAX3/BRN2 rheostat is maintained in tumours during MAPKi treatment

**Figure 6:**
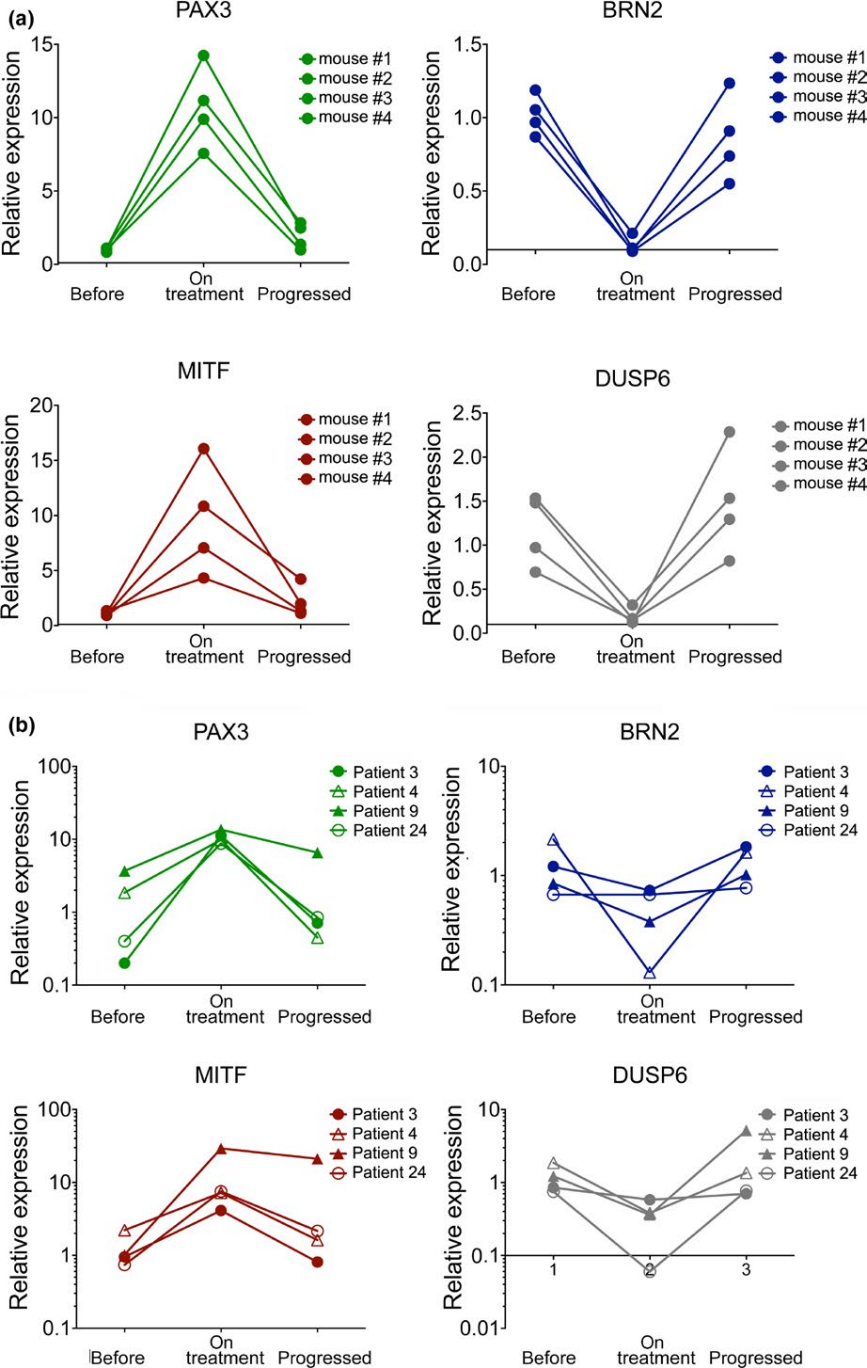
The PAX3/BRN2 rheostat can be observed in tumours from patients. (a) RT‐qPCR analysis of *PAX3*, *MITF*, *BRN2 *and *DUSP6 *expression in A375 xenografts from mice treated with vemurafenib. Samples were from before treatment, 12 days on treatment and once tumours had progressed (Smith et al., 2016). (b) RT‐qPCR analysis of *PAX3*, *MITF*, *BRN2*, and *DUSP6 *expression in melanomas from patients treated with BRAF and MEK inhibitor. Samples were from before treatment, 2 weeks on treatment and once tumours had progressed (Smith et al., 2016)

The dependence of MITF upregulation on low ERK activity suggests that in melanomas in which the PAX3/BRN2 rheostat is intact, MITF will be upregulated on treatment with MAPKi. However, if these tumours progress with ERK reactivation, the expression of PAX3, BRN2, and MITF should be restored. Indeed, we could observe these dynamics in xenografts from mice that had been treated with BRAF inhibitor (Figure 6a). Most importantly, a similar trend was observed in tumour samples from patients undergoing treatment with BRAF and MEK inhibitors (Figure 6b).

## 4 DISCUSSION

**Figure 7:**
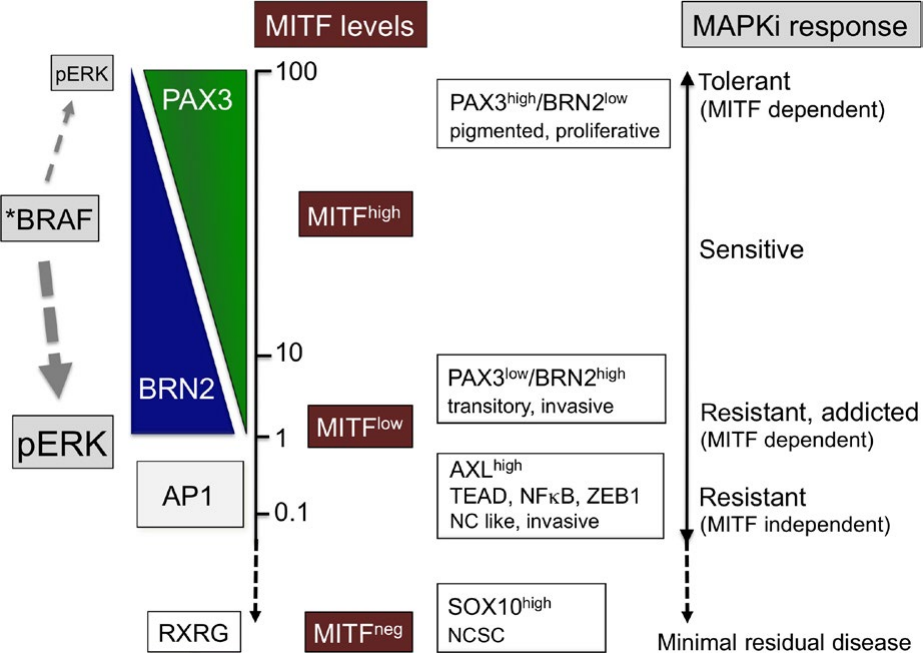
Dynamic regulation of MITF levels and their relevance to MAPKi therapy. Different melanoma cell populations can be classified by their PAX3, BRN2, MITF, and AP1 (FRA/JUN), AXL expression status. In MITF^high^ populations MITF expression is regulated through the ERK controlled PAX3/BRN2 rheostat. When ERK activity is low, PAX3 driven MITF transcription dominates leading to higher MITF expression levels; when ERK activity is high, the BRN2 contribution to transcription from the *MITF* promoter is high resulting in lower levels of MITF expression. The high levels of BRN2 also contribute to an increased invasive phenotype (Fane et al., 2018). In this PAX3^low^/BRN2^high^ population MITF still governs the melanocytic transcriptional programme (“MITF^high^”) despite low expression (“MITF^low^”), but dedifferentiated/neural crest associated traits are also present; a state that has been defined as “transitory” in a recent study defining a multi‐stage differentiation model (Tsoi et al., 2018). In MITF^low^/AXL^high^ cells MITF expression is hardly detectable, cells are dedifferentiated with regard to the melanocyte lineage and ERK activity induces an AP1 driven neutral crest (NC) and EMT like transcriptional programme, which is linked to TEAD, NFκB, and ZEB1 (Konieczkowski et al., 2014; Verfaillie et al., 2015). An even more dedifferentiated phenotype reminiscent of a neural crest stem cell (NCSC) like state is driven by RXRG, devoid of both MITF and AXL, but marked by high levels of SOX10; the population with this phenotype gives rise to minimal residual disease (Rambow et al., 2018)

MITF is essential for melanoma cells and we discovered that BRAF employs a PAX3/BRN2 rheostat to regulate MITF expression (Figure 7). Thereby, BRN2 enables BRAF to exploit constitutive PAX3 driven *MITF* transcription. By inducing BRN2 expression BRAF gains control over MITF, but by suppressing PAX3 it prevents high MITF expression, as this counteracts BRAF mediated proliferation (Wellbrock & Marais, 2005). The PAX3/BRN2 rheostat can clarify the apparently contradictory observations that ectopic BRN2 overexpression either leads to *MITF* promoter activation in A375 cells (Wellbrock et al., 2008) or suppression in 501mel cells (Goodall et al., 2008; Kobi et al., 2010). In PAX3^low^/BRN2^high^ A375 cells BRN2 is the main driver of MITF expression, and increasing BRN2 enhances this activity, but in PAX3^high^/BRN2^low^ 501mel cells overexpressed BRN2 can switch the PAX3‐driven transcription to the weaker BRN2/PAX3 driven transcription. The rheostat can also explain why depletion of endogenous BRN2 from different melanoma cell lines can result in different degrees of MITF reduction (Cook et al., 2005; Kundu et al., 2017; Simmons et al., 2017; Thurber et al., 2011; Wellbrock et al., 2008) or even an increase in MITF expression, as reported in 501mel cells (Goodall et al., 2008).

Our data support the observed existence of populations of BRN2^high^/MITF^low^ cells and vice versa (Goodall et al., 2008; Thurber et al., 2011). However, our model predicts that any intermediate state can exist and indeed in tumours BRN2 are also found significantly co‐expressed with MITF in individual melanoma cells; this can be seen in histology (Thurber et al., 2011) as well as by single‐cell gene expression analysis (Ennen et al., 2017). Furthermore, because signals from the tumour microenvironment can influence MAPK signalling the dynamic regulation of MITF downstream of BRAF might explain at least in part the significant heterogeneity in MITF expression that is observed in human tumours. Additional layers of complexity could come from PAX3 impacting on BRN2 or vice versa (Fane et al., 2018), but we did not observe any significant effects on endogenous BRN2 expression after PAX3 depletion. Nevertheless, there was a slight increase in PAX3 RNA expression when BRN2 was depleted, although this was not reflected at protein level (see Figure 3a and Supporting Information Figure S3).

Another potent MITF regulator, linked to the MITF^high^ phenotype is SOX10 (Verfaillie et al., 2015). Nevertheless, SOX10 basal expression did not correlate with basal ERK activity in our cell line panel (see Supporting Information Figure S4), and all SOX10 binding sites are outside the BRAF regulated proximal promoter region (Wellbrock et al., 2008) (see Figure 3c). Moreover, active ERK inhibits SOX10 activity (Han et al., 2018). Thus SOX10 is unlikely to contribute to the BRAF/ERK mediated basal regulation of MITF. However, in the presence of MAPKi SOX10 is active and as such, can act as an “amplifier” in our model, contributing further to MITF upregulation by enhancing PAX3 driven MITF transcription at the P2 site (Bondurand et al., 2000).

Apart from its role in the PAX3/BRN2 rheostat BRN2 has been described to impact on MITF expression through NFIB mediated upregulation of the histone methyltransferase EZH2 (Fane et al., 2017). The effect of EZH2 on MITF in the context of MAPKi is however unclear, because as BRAF/BRN2 target its expression is blocked in the presence of MAPKi. Nevertheless, when ERK reactivation occurs in resistant cells, its re‐expression could contribute to the transition to the AXL^high^ population, which displays a different transcriptional and chromatin state (Konieczkowski et al., 2014; Verfaillie et al., 2015). The regulation of MITF by MAPK signalling at this MITF^high^→AXL^high^ transition appears to be crucial. This can be seen in acquired resistant, but drug‐addicted melanoma cells, whose survival in the presence of drug is entirely dependent on the presence of MITF (Kong et al., 2017) (Figure 7). Removal of drug results in ERK hyper‐activation, MITF downregulation, and AXL upregulation. The reason for the drug addiction appears to be that cells cannot adapt to this rapid switch and are unable to “use” the new transcriptional programme driven by the AP1 factors FRA1 and JUNB (Figure 7) for survival, as this might require further epigenetic changes that take longer to establish; as a consequence cells die due to the lack of MITF.

Importantly, in cells with a fully established AXL^high^/MITF^low^‐related transcriptional programme, which is closely linked to WNT5A, TGFβ, ZEB1, NFκB, AP1, and TEAD (Figure 7), BRAF/MAPK signalling only contributes to proliferation but is not required for survival (Smith et al., 2017), possibly because other factors than MITF provide the relevant signals. Consequently, the AXL^high^/MITF^low^ phenotype is inherently MAPKi therapy resistant and enriched in progressed melanomas (Konieczkowski et al., 2014; Muller et al., 2014; Smith et al., 2017; Tirosh et al., 2016). Nevertheless, despite the focus on the AXL^high^/MITF^low^ phenotype at the centre of MAPKi resistance, recent studies revealed that there is an even more dedifferentiated phenotype reminiscent of a neural crest stem cell (NCSC) like state (Figure 7) that is devoid of MITF and AXL, but expresses high levels of SOX10 and gives rise to minimal residual disease (Rambow et al., 2018).

In summary, we found that a delicate balance between the transcription factors BRN2 and PAX3 produces great plasticity in the regulation of MITF downstream of BRAF. The PAX3/BRN2 balance is crucial for melanoma cells to maintain MITF expression levels optimal for growth (Wellbrock & Marais, 2005; Wellbrock et al., 2008; Wellbrock, Weisser, Geissinger, Troppmair, & Schartl, 2002). However, long‐term interference with this fine‐tune mechanism results in dynamic changes in MITF expression and this can pose a challenge to MAPK targeting therapy.

## CONFLICT OF INTEREST

Jennifer Wargo is a paid speaker for DAVA Oncology, Illumina and BMS and has served on advisory boards for Roche Genentech, GSK, and Novartis. All other authors declare no potential conflicts of interest.
