# Supplementary material for: A PAX3/BRN2 rheostat controls the dynamics of BRAF mediated MITF regulation in MITFhigh/AXLlow melanoma
Source: Pigment Cell Melanoma Res. 2018 Oct 19;32(2):280–91. doi: 10.1111/pcmr.12741 (PMC6392120; doi:10.1111/pcmr.12741)
Supplement: Supplementary file 1 [file PCMR-32-280-s001.pdf]

Supporting Information

A PAX3/BRN2 rheostat controls the dynamics of BRAF mediated MITF regulation in $MITF^{high}/AXL^{low}$ melanoma

Supplementary Figures

Figure S1, related to Figure 1
Correlation of PAX3 and BRN2 expression with MITF and AXL

Figure S2, related to Figure 2
Correlation of BRN2 expression with MITF and DUSP6

Figure S3, related to Figure 3
PAX3 and BRN2 regulate MITF expression

Figure S4, related to Discussion
SOX10 expression does not correlate with ERK activation

Supplementary Methods

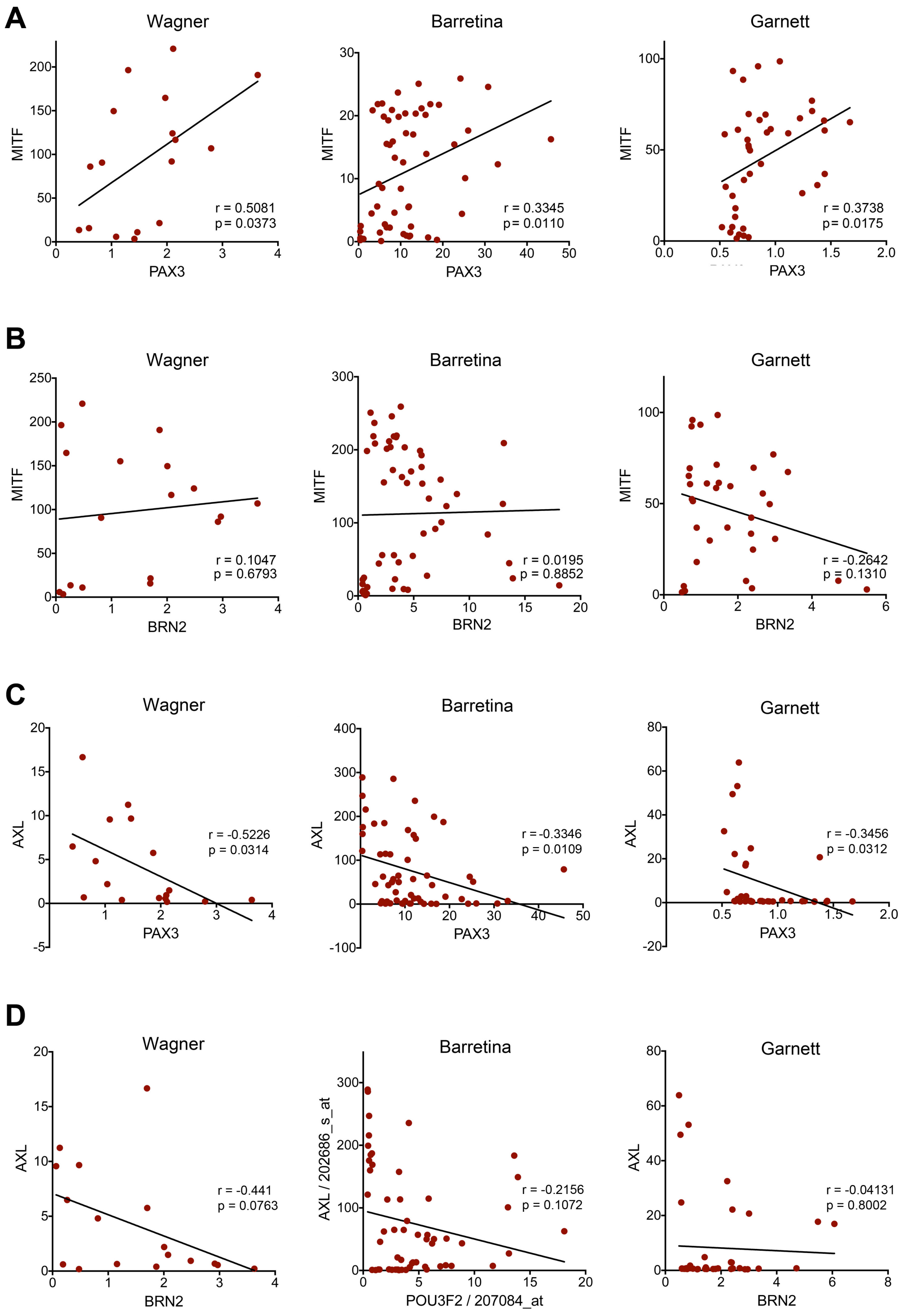

Figure S1, related to Figure 1: Correlation of PAX3 and BRN2 expression with MITF and AXL

(A) Pearson correlation analysis of MITF expression with PAX3 and **(B)** BRN2 in the Wagner, Barettina and Garnett datasets. **(C)** Pearson correlation analysis of AXL expression with PAX3 and **(D)** BRN2 in the Wagner, Barettina and Garnett datasets.

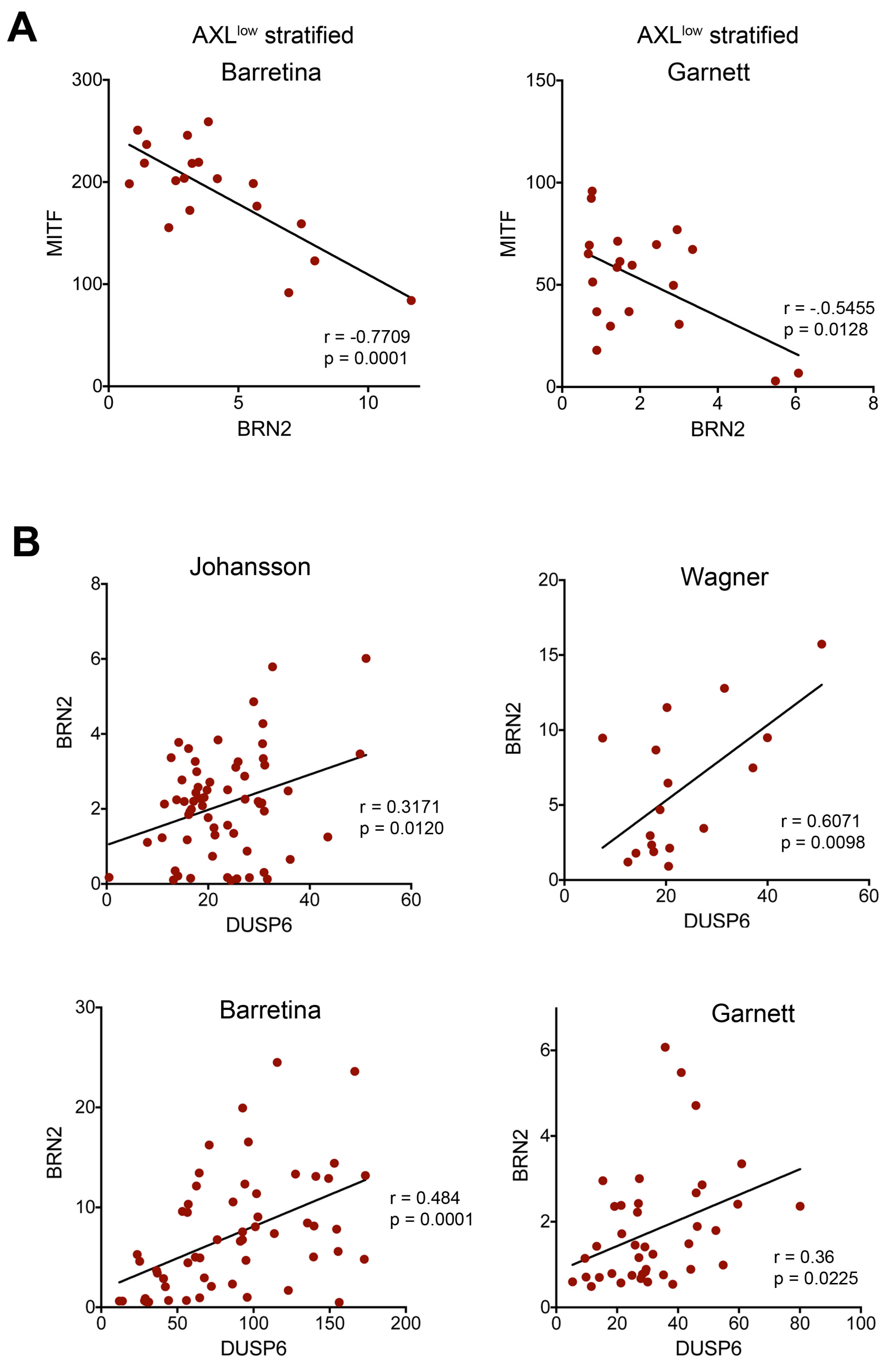

Figure S2, related to Figure 2: Correlation of BRN2 expression with MITF and DUSP6

(A) Pearson correlation analysis of MITF expression with BRN2 in AXL^{low} cells. **(B)** Pearson correlation analysis of BRN2 expression with DUSP6 in the Johansson, Wagner, Barettina and Garnett datasets.

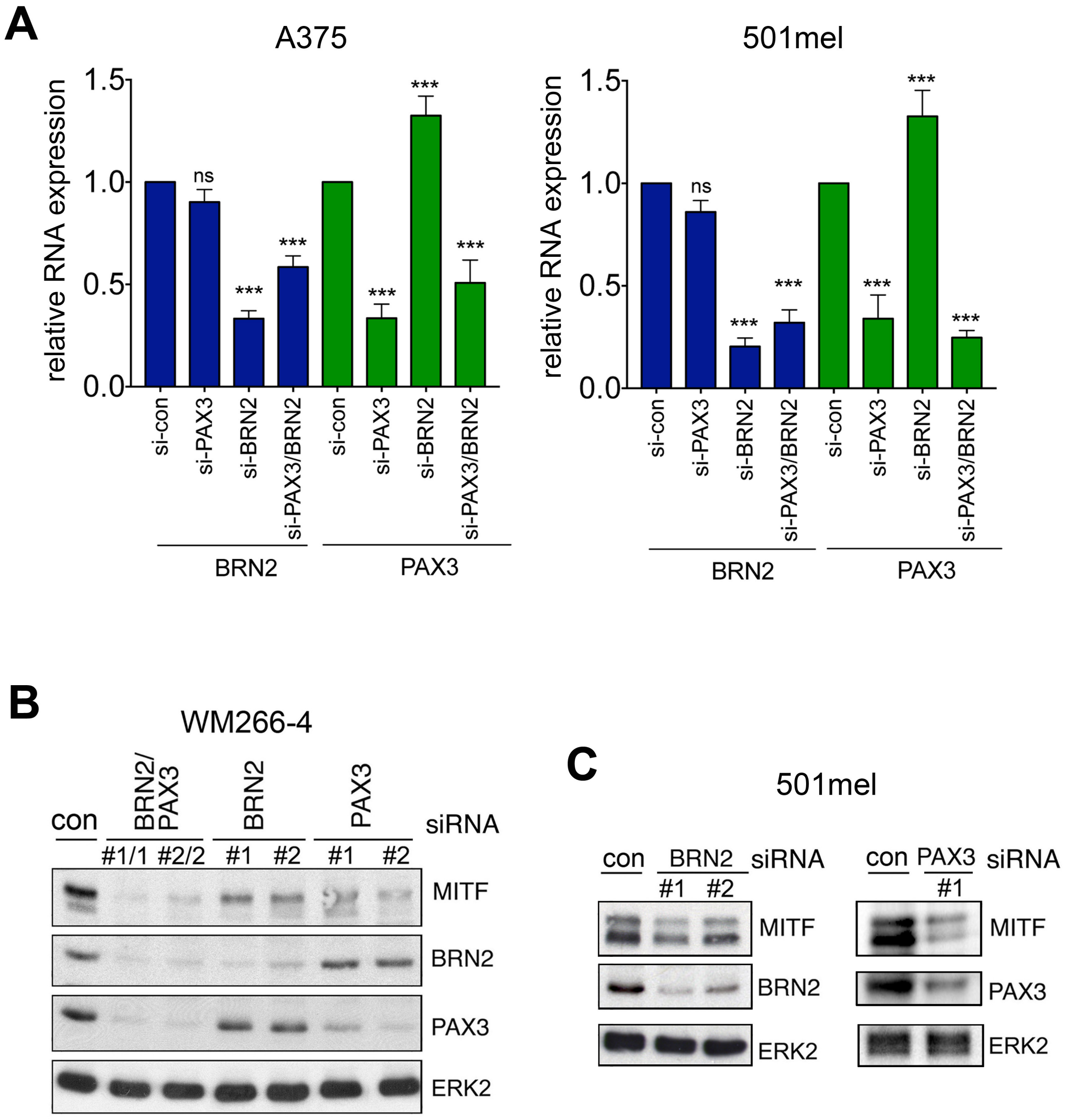

Figure S3, related to Figure 3: PAX3 and BRN2 regulate MITF expression

(A) qRT-PCR analysis of BRN2 and PAX3 expression in the indicated cell lines treated with the indicated siRNAs. **(B)** Western-blot for BRN2 and PAX3 expression in WM266-4 cells treated with the indicated siRNAs. ERK2 served as loading control. **(C)** Western-blot for BRN2 and PAX3 expression in 501mel cells treated with the indicated siRNAs. ERK2 served as loading control.

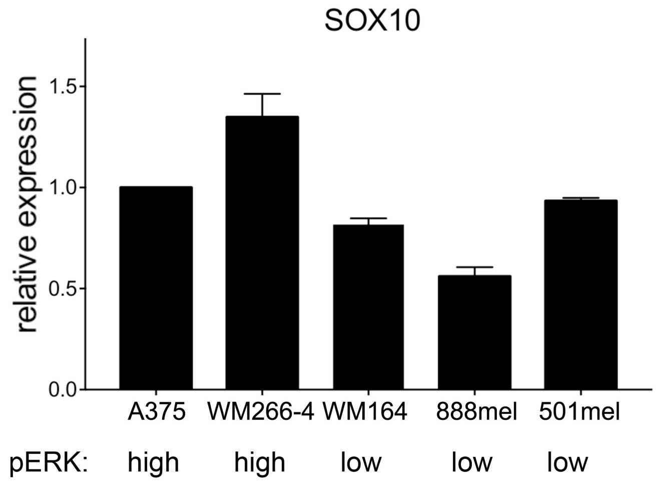

Figure S4, related to Discussion: SOX10 expression does not correlate with ERK activation

qRT-PCR for basal SOX10 expression in the indicated melanoma cell lines. The ERK-phosphorylation/activation status in the respective cell lines is indicated.

Supplementary Methods

Cell lines and transfections

A375, WM266-4, SKMel28 and SKMel2 cells were bought from the American Type Culture Collection and 501mel and 888mel cells were a gift from Steve Rosenberg (NCI, MD); all were obtained in 2008. WM164 cells were a gift from Meenhard Herlyn (Wistar Institute). All cell lines were authenticated in house by short tandem repeat profiling before and during the study; the last authentication was carried out in 2017. These cell lines were grown in DMEM/10% FCS (PAA, Yeovil, UK). Cells were transfected with plasmid DNA using Attractene (Qiagen, Valencia, CA, USA) and with siRNAs using Interferin (Polyplus, Illkirch, France).

Patient samples

Patients with mutant *BRAF*V600 positive metastatic melanoma were treated with either a BRAF inhibitor, or a combination of BRAF and MEK inhibitors (Smith et al, 2016). All patients were consented for tissue acquisition per an IRB-approved (ethical approval) protocol (Office for Human Research Studies, Dana-Farber/Harvard Cancer Center; DFCI Legacy #11-181) confirming that the study conforms to recognized standards. Tumour biopsies were obtained before treatment, on treatment and on progression.

RNA analysis, qPCR primers and siRNAS

RNA from cell lines or frozen tumour tissue was isolated with TRIZOL® as described (Smith et al., 2016). Selected genes were amplified using SYBR green (Qiagen, Valencia, CA, USA). Patient samples were pre-amplified using the TaqMan PreAmp Master Mix Kit (PN4384267, Applied Biosystems, Carlsbad, CA) according to the manufacturer's instructions. TaqMan qPCR was carried out with TaqMan Gene Expression Master Mix (PN4369016, Applied Biosystems, Carlsbad, CA).
Primer sequences for SYBR green qPCR were: PAX3: AGGATGCGGCTGATGGAACTCACTG, CCAGGATGATGCGGCCGGGCCCGGG; BRN2: TTTCCTCAAATGCCCCAAG, TTTCTGTCTC-CTGTTACAAAACCA; M-MITF: CCGTCTCTCACTGGATTGGT, TACTTGGTGGGGTTTTCGAG; DUSP6: CCGCAGGAGCTATACGAGTC, CGTAGAGCACCACTGTGTCG; BETA-ACTIN: GCAAGCAGGAGTATGACGAG, CAAATAAAGCCATGCCAATC. All siRNAs were from Dharmacon/Thermo Fisher and the sequences were: control AAUAUAAUCACUAUCAGGUGC; PAX3: #1 GAAACACCGUGCCGUCAGUUU, #2 GAGACUGGCUCCAUACGUCUU; BRN2: #1 GCGCAGAGCCUGGUGCAGGUU, #2 CCGCAGCGUCUAACCACUAUU.

Immunoblotting and antibodies

Cell lysates were analysed by standard Western-blotting protocols. The primary antibodies used were for: phospho-ERK (MAPK-YT) from Sigma, St Louis, MO, USA; ERK2 (C-14), PAX3 (N-19) and (C-20), BRN-2 (C-20) and (B-2) from Santa Cruz Biotechnolgy, Santa Cruz, CA, USA; MITF (C5) from Neomarkers, Lab Vision, Runcorn, UK.

DNA Binding Assay

Whole-cell extracts from cells transfected with pEF, pEF-BRN2 or pEF-PAX3 were prepared by four freeze-thaw cycles in low salt lysis buffer as described (Morcinek et al, 2002). Cleared supernatants were incubated with M280-Streptavidin Dynabeads (Dynal) carrying the regions from -77 to -20 of the *M-MITF* promoter. Bound proteins were eluted with 1M NaCl and analysed by Western blot.

Immunofluorescence

Samples were fixed and stained as described (Wellbrock et al, 2008). A Zeiss Axioskop2 plus was used; images were taken by a Photometrics Cool Snap HQ CCD camera driven by Metamorph software (Universal Imaging).
